# Development of a low-cost external fixation system: an off-label solution for resource-limited settings

**DOI:** 10.1007/s00068-026-03246-w

**Published:** 2026-06-16

**Authors:** Martin Seiser, Christian Deininger, Johann Fierlbeck, Marianne Hollensteiner, Patrick Coles, Caspar Reuter, Herbert Tempfer, Andreas Traweger, Florian Wichlas

**Affiliations:** 1 Paracelsus Medical University Salzburg, Salzburg, Austria; 2 Department of Orthopaedics and Traumatology, AUVA Trauma Hospital Salzburg, Salzburg, Austria; 3 Institute of Tendon and Bone Regeneration, Paracelsus Medical University, Salzburg, Salzburg, Austria; 4 Department of Orthopaedics and Trauma Surgery, Medical University of Graz, Graz, Austria; 5 Department of Technology Transfer, Paracelsus Medical University Salzburg, Salzburg, Austria; 6 Institute for Biomechanics, BG Unfallklinik Murnau, Murnau, Germany; 7 Institute for Biomechanics, Paracelsus Medical University Salzburg, Salzburg, Austria; 8 Department of Orthopaedics and Traumatology, Paracelsus Medical University, Salzburg, Salzburg, Austria

**Keywords:** Bone fractures, Biomechanical analysis, External fixator, Threaded rods, Low- and middle-income countries

## Abstract

**Purpose:**

External fixators are essential for the surgical management of long-bone fractures, particularly in resource-limited settings where access to commercial systems is often restricted by cost and logistics. This study aimed to develop a low-cost external fixation concept using widely available construction materials and evaluate its biomechanical performance under axial loading.

**Methods:**

An external fixator composed of M10 threaded rods, washers, and nuts was developed. Five configurations were tested: SP (Steinmann pins), KT (threaded K-wires), KS (smooth K-wires), and BKS (bilaterally applied, smooth K-wires). A commercial external fixator served as control (CG). Juvenile bovine ulnae with a standardized fracture gap were subjected to cyclic axial loading. Construct stiffness, elastic deformation, and plastic deformation were assessed.

**Results:**

CG showed the highest stiffness (107.80 ± 14.12 N/mm), followed by SP (87.73 ± 17.92 N/mm), BKS (83.81 ± 10.01 N/mm), KS (49.84 ± 3.80 N/mm), and KT (46.66 ± 2.39 N/mm). Post-hoc analyses showed no significant differences between CG vs. SP and CG vs. BKS, whereas KT and KS were significantly less stiff. Elastic deformation followed a similar pattern. Plastic deformation was greatest in CG, while SP and BKS showed significantly lower values.

**Conclusions:**

The threaded-rod fixator provides relevant mechanical stability at extremely low material cost. As a first biomechanical proof-of-concept, this approach may offer a practical and immediately deployable stabilization method for resource-limited settings when standard external fixators are unavailable. It could serve either as a definitive treatment or as part of a damage control strategy prior to transfer. Further biomechanical, cadaveric, and clinical studies are required before clinical application can be considered.

## Introduction

Trauma-related injuries are a major cause of disability worldwide [1–3], with low- and middle-income countries (LMICs) disproportionately affected due to high rates of road traffic accidents, occupational hazards, and conflict-related injuries [4]. LMICs and regions affected by war, face several similar challenges in management of trauma-related injuries. The high incidence of fractures in these resource-limited settings is worsened by the lack of adequate medical infrastructure, trained personnel, and affordable fixation devices, which severely limits effective treatment options [5–7].

In these settings, high-energy trauma mechanisms – including motor vehicle accidents, blast injuries, and projectile wounds – frequently result in complex fracture patterns with substantial complications. These injuries are often characterized by open fracture configurations, extensive soft tissue damage, and significant wound contamination, substantially increasing both treatment complexity and the risk of adverse outcomes such as infection and delayed union [8, 9]. Additionally, definitive treatment is frequently unavailable at the initial point of care, necessitating temporary stabilization to enable transport to higher-level facilities. This creates a critical need for stabilization methods that are both accessible and effective under austere conditions [10].

External fixators (EF) play a crucial role in the treatment of these complex injuries [10–12]. However, the widespread distribution of commercial EF systems in resource-limited settings is hindered by high costs and significant logistical barriers [13]. To address these challenges, alternative low-cost EF designs have been proposed, utilizing materials such as repurposed surgical implants, metal rods, bone cement, and even wood [14–17]. While some of these models have demonstrated promising biomechanical properties, their clinical feasibility and long-term performance remain underexplored [18]. The key challenge remains to develop solutions that combine low cost and mechanical reliability while maintaining clinical applicability.

Addressing this challenge is crucial for providing viable alternatives that meet the functional demands of trauma care in resource-limited settings. Several studies have assessed improvised EF constructs, revealing varying degrees of stiffness and stability [15, 19, 20]. Besides the biomechanical properties of alternative external fixators, there have been clinical trials with promising results [13]. However, solutions such as the imperial external fixator [13, 14] have focused on developing and implementing sophisticated low-cost alternatives to commercial systems, still requiring specialized tools and manufacturing processes.

The aim of this study was to identify the absolute minimum technical requirements for a provisional external fixation solution based on widely accessible and cost-effective standard hardware and construction components, such as threaded rods, washers, and nuts, without requiring specialized tools or manufacturing techniques. This approach addresses situations where pre-planning is impossible, such as sudden conflict escalations, natural disasters, or in remote settings. It offers an immediately implementable alternative when commercial systems are unavailable. After developing the construct, its mechanical properties are evaluated through axial compression testing to assess its basic suitability for fracture stabilization. This study provides first insights into the feasibility of a globally deployable, low-cost EF solution.

## Materials and methods

### Fixator design and construction

The developed external fixator consists of readily available materials, including M10 threaded rods (10 mm outer diameter, 8.9 mm core diameter) made of A2 stainless steel (AISI 304 equivalent, an austenitic chromium-nickel steel), M10 washers, and M10 nuts (Schraubenking GmbH, Katzenberg 58, 4982 Kirchdorf am Inn, Austria). These components were selected for their affordability, ease of procurement, and mechanical stability, making them potentially suitable for use in resource-limited settings [21]. The fixator was assembled using basic manual construction techniques to ensure worldwide producibility. All structural components are standard industrial fastening hardware, widely stocked at hardware suppliers globally, and comparable in diameter and material strength to conventional orthopaedic fixation hardware but available at a fraction of the cost.

**Fig. 1:**
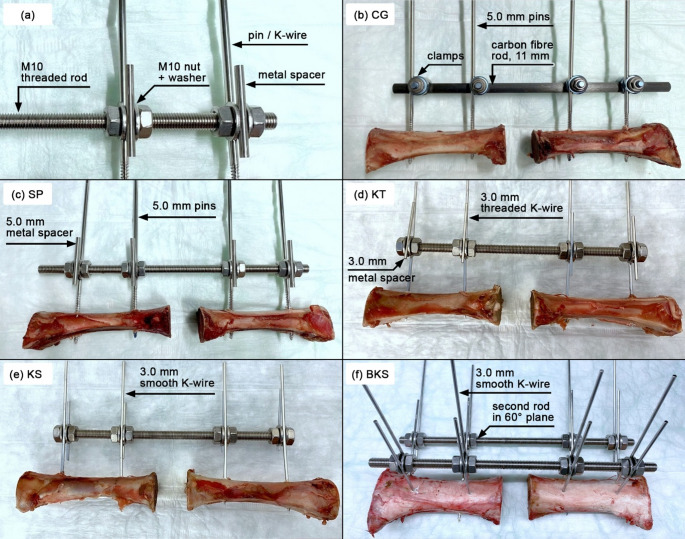
Experimental groups. (**a**) Close-up of the washer-based pin fixation mechanism; component labels apply to all threaded-rod configurations (**c**–**f**). (**b**) Control group (CG): commercial external fixator (DePuy Synthes) with 11 mm carbon fibre rod and 5.0 mm Steinmann pins. (**c**) SP: threaded-rod fixator with 5.0 mm Steinmann pins. (**d**) KT: threaded-rod fixator with 3.0 mm threaded K-wires. (**e**) KS: threaded-rod fixator with 3.0 mm smooth K-wires. (**f**) BKS: bilateral threaded-rod fixator with 3.0 mm smooth K-wires at 60° angle

Unlike conventional EF that use pre-manufactured carbon or stainless-steel rods, this design utilizes M10 threaded rods as the primary stabilization component. The fixation is achieved by inserting Steinmann pins (as in commercial fixator systems) or K-wires into the bone and securing them to the threaded rods using M10 nuts and washers. To prevent tilting of the washers during tightening and to ensure a firm grip on the bone-anchored pins, a short metal spacer of the same diameter of the inserted pins or K-wires was used (Fig. 1a). The nuts were tightened to 25 Nm using a torque wrench to ensure standardized and reproducible tightening conditions. Steinmann pins and K-wires were selected as bone-anchoring elements, as they are among the most basic and widely available orthopaedic implants, routinely used even in low-resource surgical settings [22]. All components implanted in the patient (pins and k-wires) are certified medical devices and sterile.

### Experimental groups

Five construct configurations were investigated. The control group (CG) consisted of a commercial external fixation system from DePuy Synthes, incorporating an 11-mm carbon-fibre rod and 5.0-mm Steinmann pins. To evaluate the performance of the low-cost concept, a threaded-rod fixator was applied in four alternative anchoring configurations. In the SP (Steinmann Pins) configuration, the threaded-rod fixator was combined with 5.0-mm Steinmann pins, allowing a direct comparison with the commercial system using identical pin diameters. In the KT (Threaded K-wires) configuration, the same fixator was paired with 3.0-mm threaded K-wires, whereas in the KS (Smooth K-wires) configuration smooth 3.0-mm K-wires were used to assess the influence of pin surface structure on stability. Finally, the BKS (Bilateral Smooth K-wires) configuration employed two threaded rods arranged in a bilateral 60° layout, each anchored with smooth 3.0-mm K-wires, to determine whether a dual-rod construct could compensate for the reduced stiffness associated with thinner wires.

Groups CG, SP, KT, and KS (Fig. 1b-e) were all applied in a unilateral, uniplanar configuration, with four pins or K-wires (two in the proximal and two in the distal bone fragment). The BKS group (Fig. 1f) was applied in a bilateral configuration at a 60° angle (with four K-wires per rod). An angulation of 60° lies within the anatomical safe zone defined by the AO (Arbeitsgemeinschaft für Osteosynthesefragen) at various levels of the tibia [11]. Sample sizes were 8 specimens for each group. This design allowed evaluation of the threaded rod construct across a range of anchoring options — from Steinmann pins as the most robust to smooth K-wires as the most basic and widely available — and whether threading or bilateral application can compensate for limitations associated with smooth K-wires.

### Sample preparation

**Fig. 2:**
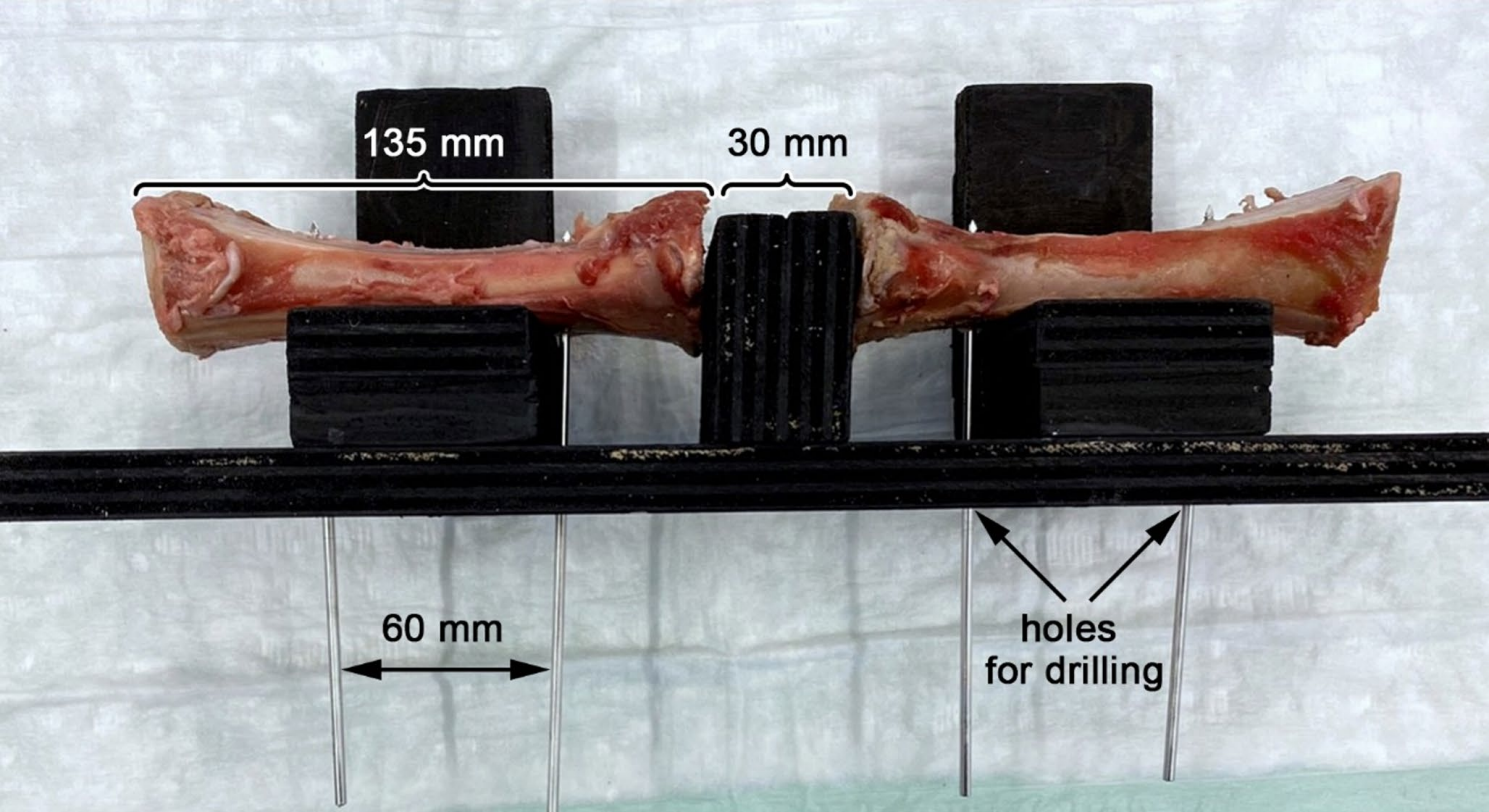
Wooden drilling template used to standardize fracture gap, pin positions, and rod-to-bone distance during specimen preparation

Juvenile bovine ulnae were used as a validated surrogate for human long bones with comparable mechanical behaviour across normal and reduced-density bone conditions [23]. The bones were cleared of soft tissue and the radius and cut into standardized lengths (134.9 mm ± 3.4 mm). To ensure consistent conditions, both ends of the bones were cut at a 90° angle to the longitudinal axis. A 30 mm fracture gap was created to simulate a comminuted fracture [24]. To maintain uniform bone positioning, a custom wooden template was used to guide pin placement and standardize the fracture gap, pin-to-pin distance, and rod-to-bone distance across all specimens (Fig. 2). This setup was used to create standardized and reproducible constructs under optimized alignment conditions. The average measured pin-to-pin distance per bone was 57.8 mm ± 1.2 mm, and the rod-to-bone distance was 40.8 mm ± 1.2 mm. All constructs were prepared and assembled by the same surgical team to minimize inter-operator variability during construct preparation.

### Biomechanical testing

**Fig. 3:**
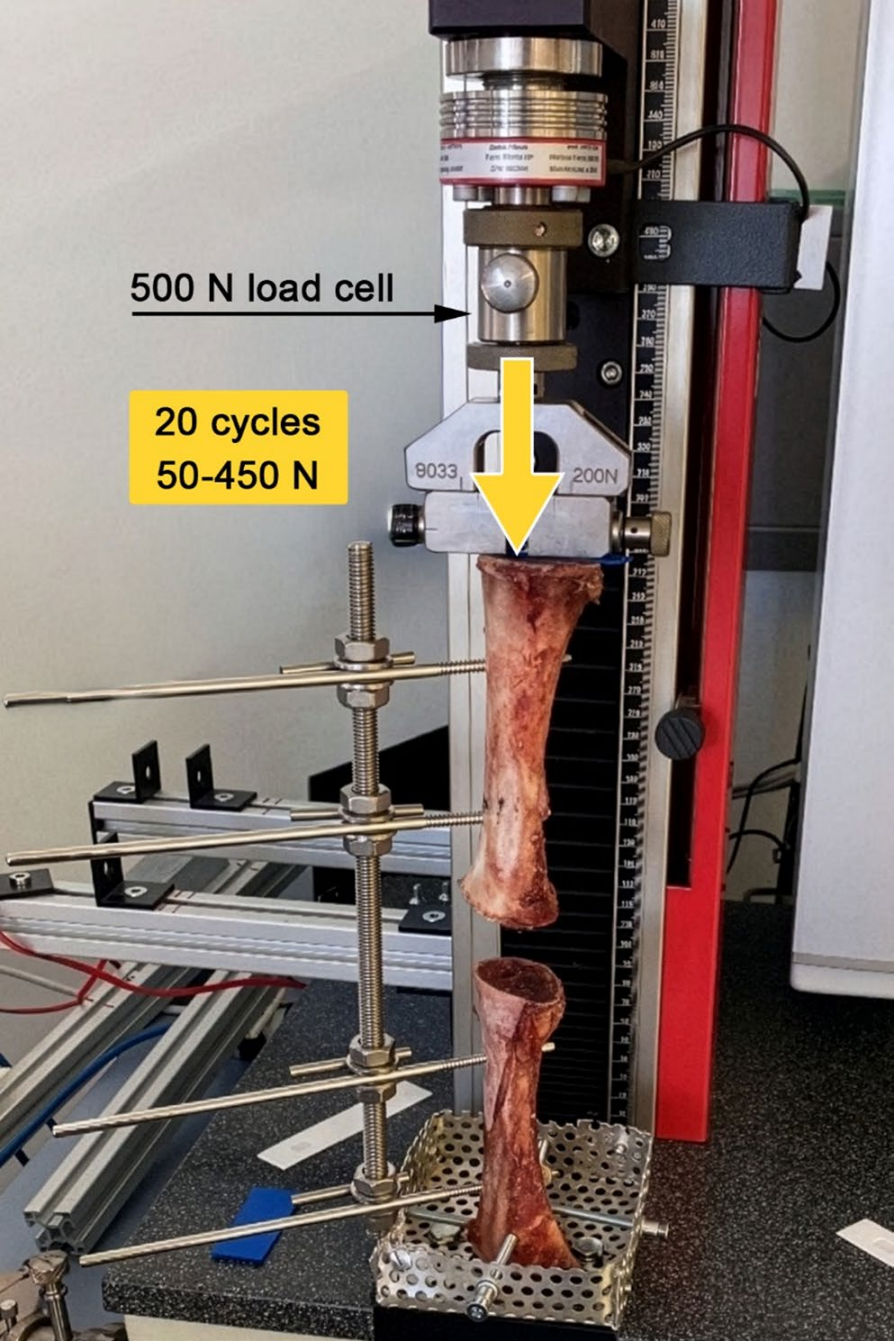
Experimental setup for axial compression testing using a universal testing machine (Zwick Roell, Type: 500 N Zwicki) with one construct in testing position

Construct stability was operationally assessed using construct stiffness as the primary outcome parameter, complemented by elastic deformation under axial loading and plastic deformation after cyclic loading. These parameters were selected to quantify resistance to axial displacement, reversible construct deformation, and residual displacement after repeated loading, respectively, and are established outcome measures in biomechanical evaluations of external fixation constructs [18, 25]. Axial compression tests were performed using a testing machine (Zwick Roell, Type: 500 N Zwicki; ZwickRoell GmbH & Co. KG, August-Nagel-Straße 11, 89079 Ulm). The constructs were positioned vertically between two parallel steel plates to ensure uniform load distribution (Fig. 3). The load was applied along the longitudinal axis of the bone-implant system to simulate physiological axial compression forces. Each construct underwent cyclic loading (valley load: 50 N, maximum load: 450 N) for 20 cycles under force control at a rate of 20 N/s. The maximum load of 450 N was selected to represent sub-physiological axial loading conditions of the tibia that occur during early postoperative partial weight-bearing [26, 27]. This load level allows discrimination of construct stiffness while avoiding premature failure of low-stiffness configurations, thereby ensuring comparability across all groups.

The first cycle was defined as a settling cycle and was not included in the final analysis. Load-displacement curves were generated using the testXpert II V3.6 software from Zwick Roell, and the construct stiffness (N/mm) was calculated as the slope of the load-displacement curve between 100 and 400 N for each test. To evaluate elastic deformation the distance moved between 50 N and 450 N was calculated for each test. Plastic deformation (i.e., residual displacement of the construct) over 20 cycles was assessed as the difference in displacement at 50 N between the first and the last loading cycle.

### Statistical analysis

No formal a priori power analysis was performed, as no prior data were available for this novel construct. The sample size of *n* = 8 per group was selected based on comparable biomechanical studies [14, 18]. Stiffness, elastic deformation and plastic deformation were analysed using the non-parametric Kruskal–Wallis test, as the effective sample sizes differed between groups due to specimen loss in the KS group (see Results) and assumptions of normality and homogeneity of variances could not be ensured. When the Kruskal–Wallis test indicated significant overall group differences, Dunn’s multiple comparisons test with adjusted p-values was used for pairwise group comparisons. All statistical analyses were performed using GraphPad Prism v. 9.02 (San Diego, CA, USA). A significance level of *p* < 0.05 was considered statistically significant.

## Results

### Construct stiffness

**Fig. 4:**
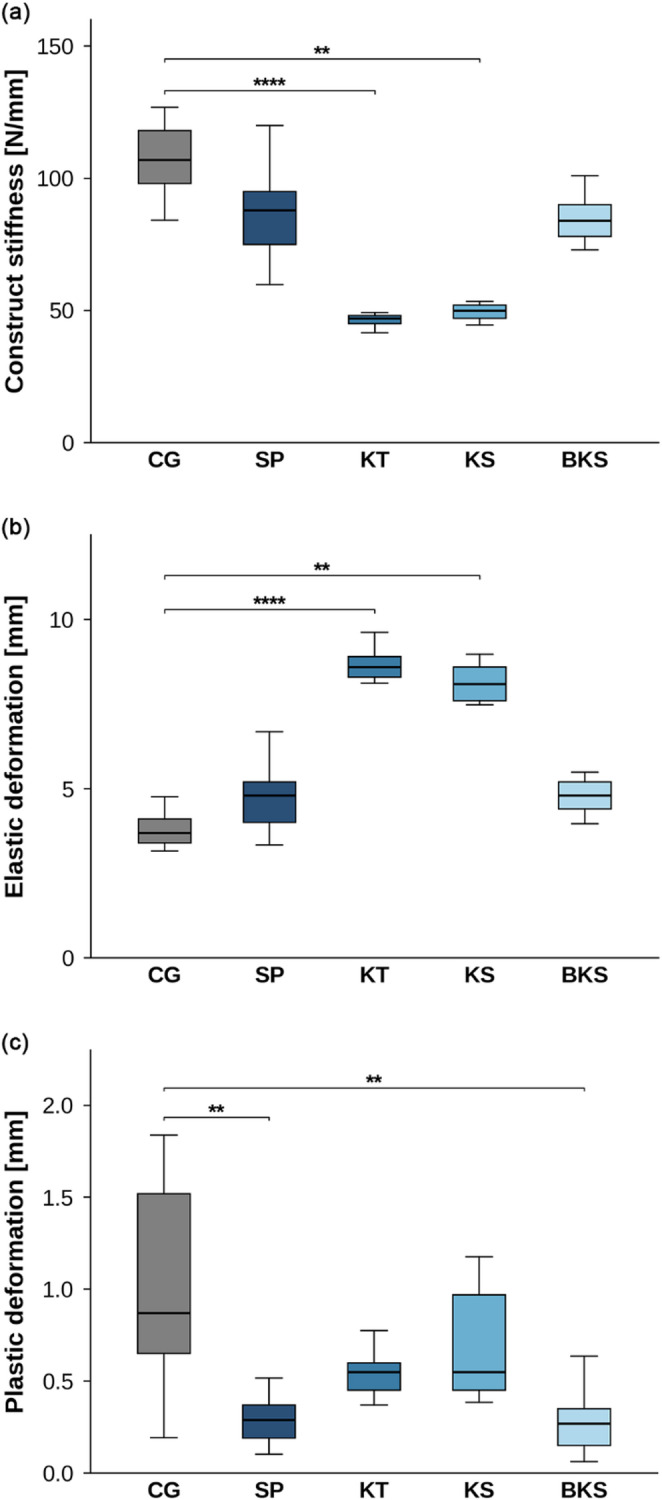
Biomechanical results for all experimental groups. (**a**) Construct stiffness (N/mm). (**b**) Elastic deformation between 50 and 450 N (mm). (**c**) Plastic deformation after 20 loading cycles (mm). Significance brackets indicate pairwise comparisons against the control group (CG) using Dunn’s test. Significance thresholds: **: *p* < 0.01; ****: *p* < 0.0001. Effective sample sizes: CG, SP, KT, BKS: *n* = 8; KS: *n* = 4

Stiffness values were different between the tested groups (Kruskal-Wallis test: *p* < 0.0001****). Construct stiffness of SP was 81.39%, BKS 77.56%, KS 46.23% and KT 43.28% of CG. Pairwise post-hoc comparison using Dunn’s test showed no significant differences between CG and SP (*p* = 0.5397) and CG and BKS (*p* = 0.1849). Stiffness values of CG compared to KS and KT were statistically significant (*p* = 0.0028** and *p* < 0.0001****, respectively). Detailed stiffness values for each experimental group are summarised in Table 1; Fig. 4a.

### Elastic deformation

Elastic deformation between 50 and 450 N differed significantly between the tested groups (Kruskal-Wallis test: *p* < 0.0001****). Compared to the control group (CG), SP showed 25.56% higher mean axial displacement, followed by BKS (28.43% higher than CG). KS and KT demonstrated higher displacement values of 113.74% and 127.84% compared to CG. Pairwise post-hoc comparison using Dunn’s test revealed no significant difference between CG vs. SP (*p* = 0.5397) and CG vs. BKS (*p* = 0.1849), whereas the difference between CG vs. KS and CG vs. KT reached statistical significance (*p* = 0.0028** and *p* < 0.0001****). Detailed values for each experimental group are listed in Table 1; Fig. 4b.

### Plastic deformation

Plastic deformation after 20 loading cycles differed significantly between the tested groups (Kruskal-Wallis test: *p* = 0.0024**). Plastic deformation was highest in CG, with the largest variability among all groups, followed by KT (63.86% of CG), KS (57.45% of CG), BKS (30.97% of CG) and SP (30.51% of CG). Dunn’s test showed no statistical significance between KT and KS compared to CG (both *p* > 0.9999). BKS and SP groups showed statistically significant lower plastic deformation values (*p* = 0.0059** for BKS and *p* = 0.0054** for SP). Detailed values of plastic deformation are listed in Table 1; Fig. 4c.

### Incomplete tests

In the KS group four of eight tests (50%) were validly completed. The remaining four tests were aborted due to dislocation of the smooth K-wires from the bone fragment during the settlement cycle, before any load cycles could be performed. No such failures occurred in any other group (SP: 0/8, KT: 0/8, BKS: 0/8, CG: 0/8). The dislocated specimens were excluded from the biomechanical analysis, as no load-displacement data could be obtained. The effective sample size for the KS group was therefore *n* = 4.

**Table 1:** Biomechanical results for all experimental groups. Values are mean ± SD

Group	*n*	Stiffness (*N*/mm)	Elastic deformation (mm)	Plastic deformation (mm)
CG	8	107.80 ± 14.12	3.77 ± 0.52	0.95 ± 0.57
SP	8	87.73 ± 17.92	4.75 ± 1.00	0.29 ± 0.14
KT	8	46.66 ± 2.39	8.59 ± 0.47	0.55 ± 0.14
KS	4*	49.84 ± 3.80	8.06 ± 0.64	0.61 ± 0.38
BKS	8	83.81 ± 10.01	4.84 ± 0.55	0.29 ± 0.18

* Four specimens excluded due to K-wire dislocation during the settlement cycle (see Results)

## Discussion

This study demonstrates that the mechanical performance of the threaded-rod fixator strongly depends on pin type and configuration. While SP and BKS setups showed stiffness values close to those of the commercial fixator, constructs using thinner K-wires (KT and KS) demonstrated markedly reduced stability, consistent with previous findings linking pin diameter to frame stiffness [11, 28]. Nevertheless, the system enables flexible use of available materials: thicker Steinmann pins significantly increase stiffness, and bilateral application – such as in the BKS configuration – can partially compensate for reduced rigidity in thinner-pin constructs. We therefore recommend using the largest available diameter and, where necessary, reinforcing the frame by adding a second rod or applying a bilateral layout. Depending on the fracture characteristics, constructs with lower stiffness may still suffice – particularly in the upper extremity or in temporary damage control situations but may require early-phase partial or non-weight-bearing.

The addition of a second stabilisation rod at 60° in the BKS configuration partially compensates for the loss of stiffness seen in unilateral K-wire setups. This setup showed an improvement of 67.76% in stiffness over unilateral K-wire configurations, although it remained 22.44% lower than modular fixator systems with carbon rods and adjustable clamps (CG). This finding aligns with previous work showing that bilateral configurations enhance frame rigidity and reduce interfragmentary movement, potentially improving fracture healing outcomes [11, 29].

Elastic displacement varied considerably between configurations. SP and BKS showed values close to the commercial fixator, whereas KT and KS demonstrated almost double the displacement. The clinical relevance of absolute displacement values depends on fracture characteristics. While limited interfragmentary motion may promote callus formation and excessive movement may impair healing [30, 31], precise thresholds are not established. Therefore, the markedly higher displacement in KT and KS should be interpreted with caution, as such increased motion may be clinically relevant depending on the fracture scenario.

All constructs showed some degree of permanent deformation after 20 loading cycles. Interestingly the commercial fixator demonstrated the highest degree of plastic deformation, while SP and BKS showed the lowest values. Although the limited number of cycles restricts conclusions about long-term fatigue behaviour, the low plastic deformation in SP and BKS indicates no tendency toward increased permanent deformation under repetitive axial loading of the threaded-rod fixator compared to the commercial fixator.

Notably, in the KS group, four of eight specimens could not be tested due to dislocation of the smooth K-wires during the settlement cycle. This finding highlights a clinically relevant limitation of smooth K-wires as anchoring elements: unlike threaded K-wires, smooth wires lack mechanical interlock with the bone and may migrate under even minimal loading. The absence of similar failures in the KT group — which used threaded K-wires of identical diameter — suggests that the thread provides sufficient purchase to resist pull-out. From a clinical perspective, the use of smooth K-wires as sole fixation elements in this construct cannot be recommended without additional measures to prevent wire migration. Threaded K-wires should be preferred when available, as the thread provides sufficient purchase to resist pull-out [32]. If only smooth K-wires are available, a bilateral configuration (as in the BKS group, where no failures occurred) may mitigate this risk by distributing forces across a larger number of anchoring points.

One of the most compelling advantages of this system is its extremely low cost. All components are standard industrial hardware and can be purchased at minimal cost in many regions [21]. Compared to commercial systems, which may cost several hundred dollars, the material cost of our construct was less than three dollars per unit, making it a viable solution for LMICs and crisis zones.

Despite the promising findings, several limitations must be acknowledged. Handling during application is less user-friendly than with commercial systems, and the lack of three-dimensional adjustability reduces intraoperative flexibility. Moreover, each pin or K-wire must be manually secured with nuts and washers, potentially increasing operative time and reducing reproducibility, particularly in settings with limited surgical training. Unlike commercial systems with adjustable clamps that can accommodate angular deviations, the washer-based fixation relies on parallel pin alignment, making accurate pin placement more critical for construct stability. Additionally, the maximum applied axial load of 450 N does not reflect full weight-bearing forces of the lower extremity [27], implying that clinical use would require reduced loading such as partial or non-weight-bearing in the early phase.

The biomechanical results of this study are based on standardized A2 stainless steel components sourced in Europe. In resource-limited settings, the available steel grade and quality may differ, potentially affecting construct stiffness and corrosion resistance. However, the fixator concept is designed to be adaptable to locally available materials and can be modified accordingly — for example, by using larger rod diameters or bilateral configurations to compensate for lower material stiffness.

In addition to these clinical and design-related considerations, several methodological factors also limit the interpretation of our results. The standardized and optimized assembly of the devices used in this study may overestimate performance under real-world clinical conditions, where angular or positional deviations during pin insertion are likely and could reduce device stiffness or increase the risk of slippage. Furthermore, since all devices were assembled by a single surgical team, it was not possible to assess inter-surgeon variability in the handling and assembly of the devices.

Although juvenile bovine ulnae are validated surrogates for human long bones in biomechanical testing [23], all soft tissues were removed prior to testing, eliminating the stabilising and damping effects that muscles, fascia, and periosteal structures provide in vivo.

Biomechanical testing was limited to axial compression. While axial stiffness is an important parameter for construct evaluation, physiological loading of the tibia during gait also includes bending and torsional components [27], which were not assessed. As a result, the present results may overestimate construct performance under multidirectional loading conditions.

Taken together, these limitations underline that the present work represents a first biomechanical proof-of-concept. Future research should therefore expand the biomechanical evaluation of the fixator beyond axial loading to include torsional and bending tests, as well as long-term cyclic fatigue testing over several thousand cycles. Cadaveric studies incorporating soft tissue envelopes are needed to assess anatomical fit, construct stability, and surgical handling under more realistic clinical conditions. Further work could also investigate the influence of different rod diameters, multi-rod or hybrid configurations, and modified clamping strategies — such as toothed or spring washers to improve pin grip or wing nuts to enable tool-free assembly — that may improve practicality while preserving the low-cost and low-tech nature of the design. In addition, studies involving a broader group of surgeons will be necessary to evaluate reproducibility and ease of application. Finally, implementation research should explore local supply chains, training requirements, and integration of the concept into treatment pathways for resource-limited or crisis settings.

The use of M10 threaded rods and standard hardware components ensures excellent accessibility and cost-effectiveness, as these materials are widely available even in low-resource environments. Their corrosion-resistant A2 stainless steel composition allows standard autoclave sterilization and reuse, supporting deployment in settings with unreliable supply chains. The appropriate sterile handling protocol for the non-implanted components would need to be determined at the point of care based on local resources and conditions. Unlike commercial modular systems that require pre-manufactured clamps and connectors, the simplicity of the developed construct enables local production and on-site customization using basic tools. These characteristics position the threaded-rod fixator as a practical and scalable option for emergency fracture management in resource-limited and crisis settings.

## Conclusion

This study demonstrates that a low-cost external fixator constructed from widely available materials can provide relevant mechanical stability. The SP and BKS configurations showed promising results in terms of stiffness and deformation resistance, making them viable alternatives to commercial EF systems in resource-limited settings.

The findings indicate that unilateral configurations using K-wires exhibit reduced stiffness compared to Steinmann pin constructs. Additionally, smooth K-wires showed a high rate of wire dislocation, rendering them unreliable as sole anchoring elements. The addition of a second stabilisation rod (BKS) compensated for both the reduced stiffness and the risk of wire migration, representing a feasible option in cases where Steinmann pins are unavailable.

By providing the first biomechanical proof-of-concept of an accessible and adaptable external fixator, this study contributes to the ongoing efforts to improve fracture care in resource-limited settings. Implementing locally manufactured, low-cost EF solutions have the potential to expand surgical treatment options and improve patient outcomes in underserved regions.

## Data Availability

The datasets generated and analysed during the current study are available from the corresponding author upon reasonable request.
